# Iron Adequacy Modifies the Association Between Prenatal Diet Quality and Pregnancy‐Related Anxiety in Pregnant Women: A Cross‐Sectional Study

**DOI:** 10.1002/fsn3.72308

**Published:** 2026-09-08

**Authors:** Ezgi Arslan Yuksel, Tuba Kahraman, Beyza Sisman, Lara Hidimoglu, Ahmet Murat Gunal

**Affiliations:** ^1^ Faculty of Health Science, Nutrition and Dietetics Halic University Istanbul Turkey

**Keywords:** diet quality, iron adequacy, pregnancy, prenatal dietary assessment

## Abstract

Diet quality may influence maternal psychological well‐being through nutrients involved in neurotransmitter synthesis and brain energy metabolism. Iron may be particularly relevant because its requirements increase during pregnancy; however, whether dietary iron adequacy modifies the association between prenatal diet quality and pregnancy‐related anxiety remains unclear. This study examined the association between prenatal diet quality and pregnancy‐related anxiety and whether dietary iron adequacy moderated this relationship. This cross‐sectional study included 293 pregnant women in Istanbul, Türkiye (mean age: 29.60 ± 4.29 years). Prenatal diet quality was assessed using the Prenatal Diet Quality Index (PDQI), anxiety using the Pregnancy‐Related Anxiety Scale (PRAS), and dietary iron adequacy as the percentage of the Recommended Dietary Allowance met through dietary intake. Moderation analysis was performed using multiple linear regression adjusted for sociodemographic and obstetric covariates. The mean PRAS score was 70.44 ± 16.07 on a scale of 33–132, and the mean PDQI score was 42.76 ± 9.73 out of 80. As no established cut‐off values were available, scores were not classified as high or low. PDQI and dietary iron adequacy were not independently associated with anxiety; however, their interaction was significant (*B* = −2.501, *p* = 0.007). Higher diet quality was associated with lower anxiety only among women with relatively high dietary iron adequacy. Maternal age, income, and planned pregnancy were also independently associated with anxiety (*p* < 0.001). The association between prenatal diet quality and pregnancy‐related anxiety may vary according to dietary iron adequacy. However, as iron was the only nutrient‐specific moderator assessed and biochemical iron status was not measured, the findings do not establish a unique or causal role for iron. Future longitudinal studies should evaluate multiple micronutrients using biomarker‐based measures.

## Graphical abstract

Higher prenatal diet quality was associated with lower pregnancy‐related anxiety, but this association was evident primarily among women with higher dietary iron adequacy. These findings suggest that adequate iron intake may play an important role in strengthening the potential relationship between overall diet quality and maternal psychological well‐being during pregnancy.

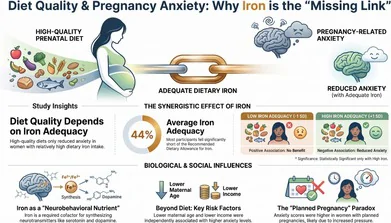

## 1 Introduction

Diet quality before and during pregnancy is critically important for maternal and child health (Deierlein et al. 2021; Doyle et al. 2016). Adequate intake of energy and nutrients supports the growth of maternal–fetal tissues and contributes to the formation of nutrient reserves for the lactation period (Doyle et al. 2016; Fowles et al. 2012). Inadequate nutrition can lead to maternal complications such as gestational diabetes and hypertension and may also result in adverse fetal outcomes (Deierlein et al. 2021; Nathanson et al. 2018; Doyle et al. 2016). Therefore, assessing diet quality during pregnancy in the context of recommendations for iron, folate, and other essential nutrients is crucial (Deierlein et al. 2021). However, existing evidence shows that many women struggle to meet dietary standards during the prenatal period (i.e., from conception to birth) (Savard et al. 2019; Nathanson et al. 2018; Han et al. 2015).

Diet quality is a comprehensive concept that captures the overall dietary pattern of individuals and is assessed through various indices developed to evaluate adherence to dietary guidelines, food diversity, or health‐promoting dietary patterns (Doyle et al. 2016; Han et al. 2015). Considering the complex and synergistic effects of nutrients during pregnancy, the relationship between diet quality and perinatal outcomes should be examined using an overall dietary quality approach (Fowles et al. 2012). Accordingly, essential micronutrients play a critical role in preventing adverse maternal and perinatal health outcomes. Pregnancy is a period of increased nutritional vulnerability due to elevated nutrient requirements. Consequently, inadequate micronutrient intake is common, particularly among women with poor overall diet quality (Cano‐Ibáñez et al. 2020).

Among these micronutrients, iron is of particular importance not only because of its role in oxygen transport, but also because it is increasingly recognized as a neurobehavioral nutrient (Georgieff 2020). Iron is a cofactor for key enzymes involved in the synthesis of monoamine neurotransmitters such as serotonin and dopamine; therefore, iron deficiency may impair neurotransmitter metabolism, hormonal balance, and brain oxygenation, which may negatively affect mood and cognitive function (Zych‐Krekora et al. 2025; Georgieff 2020). In addition, the fetal brain is a highly metabolic organ, and iron is required for ATP production and mitochondrial function. Iron deficiency during fetal and early life periods has been shown to impair hippocampal development, synaptic plasticity, and memory function (Zych‐Krekora et al. 2025; Georgieff 2020). These findings suggest that iron deficiency may influence not only hematological outcomes but also neurodevelopment and emotional regulation, including anxiety‐related outcomes (Zych‐Krekora et al. 2025).

Evidence from clinical populations outside pregnancy also suggests that dietary diversity may be associated with psychological well‐being. In a hospital‐based study conducted among Bangladeshi patients with Type 2 diabetes mellitus during the COVID‐19 pandemic, low dietary diversity was associated with higher odds of anxiety and depressive symptoms, alongside sociodemographic, lifestyle, and healthcare access‐related factors (Hossain et al. 2024). Given the differences in population characteristics and the pandemic‐related context, these findings cannot be directly generalized to pregnant women; nevertheless, they support the broader premise that dietary patterns and psychological health may be interrelated.

Although diet quality and dietary iron adequacy have been studied separately in relation to maternal mental health, it is possible that overall diet quality alone may not be sufficient to influence psychological outcomes unless micronutrient adequacy is achieved. This potential synergy suggests that a high‐quality dietary pattern may provide a metabolic environment—rich in essential cofactors like vitamin C—that serves as a promoter of iron absorption (Sitorus et al. 2024). From a nutritional perspective, diet quality reflects overall dietary pattern, whereas micronutrient adequacy reflects nutrient‐specific sufficiency. Whether dietary iron adequacy exerts a moderating effect on the relationship between overall diet quality and pregnancy‐related anxiety remains unexplored in the current literature. Notably, this interaction has not been sufficiently examined in pregnant populations. Therefore, this study aimed to examine the association between prenatal diet quality and pregnancy‐related anxiety and to determine whether dietary iron adequacy modifies this association in pregnant women.

## 2 Participants, Ethics and Methods

### 2.1 Participants

The study power was calculated using G*Power software (version 3.1.9.2, Düsseldorf, Germany), based on effect size estimates reported in previous studies investigating the association between prenatal diet quality and pregnancy‐related outcomes, assuming a Type I error of *α* = 0.05, a Type II error of *β* = 0.20, and a statistical power of 80% (Faul et al. 2007).

Participants were recruited voluntarily, and data were collected via interviewer‐administered online surveys. To ensure data quality and standardize the collection process, all participants underwent video‐based interviews conducted by the researchers after providing informed consent. Self‐reported information obtained from participants included maternal demographic and reproductive characteristics such as age, education level, occupation, marital status, household income, parity, trimester, planned pregnancy, fertility treatment history, and vitamin–mineral supplement use. Participants were also asked about chronic medical conditions diagnosed before pregnancy (including diabetes, hyperlipidaemia, hypertension, gastrointestinal disorders, and psychiatric conditions) and any new diagnoses made during pregnancy.

A total of 325 pregnant women were invited to participate in the study. Of these, 310 agreed to participate. After applying the eligibility criteria and excluding 17 women due to incomplete questionnaire and/or dietary assessment data, the final analytical sample consisted of 293 pregnant women. Only participants with complete questionnaire and dietary assessment data were included in the analyses; therefore, no additional completion threshold was applied.

### 2.2 Measurements

Participants attended a single study visit. Anthropometric measurements, including pre‐pregnancy body weight (kg), current body weight (kg), and height (cm), were obtained from all participants. Body mass index (BMI) was calculated using these measurements. Current gestational weight gain was calculated as the difference between self‐reported pre‐pregnancy body weight and current body weight measured at the time of study assessment. Therefore, gestational weight gain reflects weight gain up to the participant's current gestational age rather than total weight gain throughout pregnancy. To assess prenatal diet quality, three non‐consecutive 24‐h dietary recalls were obtained from the pregnant women. The dietary records were analyzed using the Nutrition Information System software, Istanbul, Turkiye (BeBiS 8.0 2021).

Pregnancy‐related anxiety was assessed using the Pregnancy‐Related Anxiety Scale (PRAS). The scale was originally developed by Brunton et al. in 2019 (Brunton et al. 2019), and its Turkish cultural adaptation, validity, and reliability study was conducted by Şolt Kırca and Kanza Gül in 2020 (Şolt and Gül 2020). Although no established cut‐off score is available for the PRAS, previous studies have reported mean scores ranging from 29.40 to 57.07 in pregnant populations, providing context for interpreting the observed scores (Geusens and Skalkidou 2025; Samra and Dryer 2024).

The PRAS is a 33‐item, 4‐point Likert‐type scale, including 11 reverse‐scored items. Total scores range from 33 to 132, with higher scores indicating greater pregnancy‐related anxiety. The scale consists of nine subscales: Childbirth Concerns (six questions), Body image concerns (five questions), Attitudes Toward childbirth (three questions), Worry About Motherhood (three questions), Acceptance of Pregnancy (three questions), Anxiety indicators (four questions), Attitudes Toward Medical Staff (three questions), Avoidance (three questions) and Baby Concerns (three questions). The scale does not have a clinical cut‐off point. The original PRAS reported Cronbach's alpha values between 0.77 and 0.95. In the present study, Cronbach's α was 0.85.

In addition, prenatal diet quality was assessed using three non‐consecutive 24‐h dietary recalls obtained from the pregnant women and the Prenatal Diet Quality Index (PDQI), which was developed by Bodnar and Siega‐Riz in 2002 (Bodnar and Siega‐Riz 2002). The PDQI consists of eight components: the percentage of recommended daily servings for grains, vegetables, and fruits; the percentage of the Recommended Dietary Allowance (RDA) for folate and iron; the percentage of Adequate Intake (AI) for calcium; the percentage of total calories from fat; and a meal pattern score. The first three components reflect dietary adequacy for grains, vegetables, and fruits based on the Dietary Guidelines and Food Guide Pyramid (Shaw et al. 1998). The next three components capture intake of nutrients particularly important during pregnancy—folate, iron, and calcium. These nutrients represent intake from diet alone, excluding supplemental sources. The seventh component reflects the percentage of total energy derived from fat, while the final component assesses meal and snack patterns (Institute of Medicine, and Guide, S. for a C. A 2009).

Each component was converted to a score ranging from 0 to 10, yielding a total PDQI score ranging from 0 to 80, with higher scores indicating better prenatal diet quality. All eight components contributed equally to the total score (Bodnar and Siega‐Riz 2002). Equal weighting was applied because there is insufficient evidence to determine the relative impact of individual dietary factors on pregnancy or birth outcomes (Bodnar and Siega‐Riz 2002). A score of “0” was assigned if a specific food group was not consumed, while a score of “10” was assigned if the intake met or exceeded recommended servings; intermediate scores were calculated proportionally (Octavia et al. 2020).

Grain, vegetable, and fruit intake was converted into scores based on the portion recommendations of the Turkish dietary guideline, the Türkiye Nutrition Guide (TUBER), developed with the support of the Ministry of Health (TUBER, 2022). Dietary folate intake was expressed as dietary folate equivalents (DFE). DFE was calculated using naturally occurring food folate and folic acid from fortified foods only, using the following formula:

DFE = (1.7 × folic acid (μg)) + folate (μg) (Bailey 2000).

Folic acid and folate values were obtained from the BeBIS (8.0) nutrient database. Dietary folate, iron, and calcium intakes were then calculated as percentages of the RDA appropriate for maternal age and pregnancy status. These variables reflect dietary intake rather than biochemical nutrient status and should not be interpreted as a biomarker‐based measure of nutrient sufficiency. According to TUBER (2022), the recommended daily intakes are as follows:

- Folate: 600 μg (pregnant women) versus 330 μg (women of reproductive age)
- Iron: 16–27 mg (pregnant women) versus 16 mg (women of reproductive age)
- Calcium: 950–1000 mg (for both pregnant and reproductive‐age women)

These percentages were subsequently converted into PDQI nutrient component scores. In the PDQI, total fat was defined as the percentage of energy derived from fat, and an intake of ≤ 30% of total energy from fat received the maximum score. The highest score for meal pattern was assigned to individuals consuming three meals and two snacks per day (Bodnar and Siega‐Riz 2002).

To assess prenatal diet quality, three non‐consecutive 24‐h dietary recalls (two weekdays and one weekend day) were collected within the same week by a trained research dietitian using the multiple‐pass recall method. Average daily nutrient intakes were calculated from the three recalls and analyzed using the Nutrition Information System software (BeBIS 8.0, Istanbul, Türkiye). BeBIS integrates the German Food Code and Nutrient Database (BLS), the USDA food composition database, and the Turkish National Food Composition Database (TürKomp), together with Turkish traditional foods and commercially available food products (BeBIS 8.0, Istanbul, Türkiye). Based on the BeBIS outputs, the daily PDQI score was calculated in a Microsoft Excel database, and an average score was computed for each participant for further data analysis (Fowles et al. 2012).

### 2.3 Ethical Considerations

Ethical approval for human research was obtained from the Haliç University Non‐Interventional Clinical Research Ethics Committee (no. 2025/294), and informed voluntary consent was obtained from all participants. Confidentiality was ensured through the de‐identification of personal and location data.

### 2.4 Statistical Analysis

Data were analyzed using SPSS version 24.0 (SPSS Inc., Chicago, IL, USA). Descriptive statistics were presented as frequencies and percentages for categorical variables, and as mean ± standard deviation along with minimum–maximum values for continuous variables. To compare Pregnancy‐Related Anxiety Scale (PRAS) scores, Prenatal Diet Quality Index (PDQI) scores, and dietary iron adequacy (% of Recommended Dietary Allowance, RDA) across trimesters, a one‐way analysis of variance (ANOVA) was performed. Effect sizes were reported using eta‐squared (*η* ^2^). Since ANOVA results were non‐significant, post hoc analyses were not conducted. Multiple linear regression analysis was performed to identify factors associated with pregnancy‐related anxiety. The regression model was adjusted for potential confounders, including maternal age, pre‐pregnancy body mass index (BMI), education level, income level, trimester, planned pregnancy, fertility treatment, pre‐pregnancy disease status, and parity. Model fit was evaluated using *R* ^2^, adjusted *R* ^2^, F statistics, and *p*‐values. Multicollinearity was assessed using variance inflation factors (VIF), and no multicollinearity issues were detected (VIF < 2). The moderating effect of dietary iron adequacy on the relationship between PDQI and PRAS scores was examined using standardized variables (Z‐PDQI and Z‐dietary iron adequacy). An interaction term (PDQI × dietary iron adequacy) was included in the regression model to test the moderation effect. The significance of the interaction term and its contribution to the explained variance were used to evaluate the moderating effect. A significance level of *p* < 0.05 was used for all statistical analyses.

## 3 Results

The demographic characteristics of the participants are summarized in Table 1. A total of 293 healthy pregnant women with a mean age of 29.60 ± 4.29 years participated in our study. Approximately two‐thirds of the pregnant women had undergraduate degrees (67.2%, *n* = 197) and only 4.8% of pregnant women had postgraduate degrees. In addition, the majority of participants were in the second trimester (53.9%, *n* = 158), while the lowest proportion was observed in the first trimester (8.2%, *n* = 24). The mean pre‐pregnancy BMI was 24.30 ± 2.99 kg/m^2^ and the current gestational weight gain averaged 7.47 ± 3.49 kg. Additionally, approximately half of the participants had two or more previous births (48.5%, *n* = 142) and only 9.2% of pregnant women had received fertility treatment. Regarding household annual income, 64.2% of the pregnant women were in the middle‐income group and 23.5% were in the high‐income group, and 67.9% of the pregnancies were planned. The mean anxiety score of the participants was 70.44 ± 16.07, the diet quality index (PDQI) score was 42.76 ± 9.73 (possible range: 0–80), and the percentage of dietary iron adequacy was 44.08 ± 17.71.

**TABLE 1:** Maternal demographic, obstetric and clinical characteristics of the participants (*n* = 293).

Variable	Value
Maternal age (years)	29.60 ± 4.29 (19.00–42.00)
Maternal education, *n* (%)	
Primary school	3 (1.0%)
Secondary school	9 (3.1%)
High school	49 (16.7%)
College	21 (7.2%)
Undergraduate degree	197 (67.2%)
Postgraduate degree	14 (4.8%)
Trimester, *n* (%)	
1st trimester	24 (8.2%)
2nd trimester	158 (53.9%)
3rd trimester	111 (37.9%)
Income, *n* (%)	
Low	36 (12.3%)
Middle	188 (64.2%)
High	69 (23.5%)
Planned pregnancy, *n* (%)	
Yes	199 (67.9%)
No	94 (32.1%)
Fertility treatment, *n* (%)	
Yes	27 (9.2%)
No	266 (90.8%)
Marital status, *n* (%)	
Married	285 (97.3%)
Not married	8 (2.7%)
Pre‐pregnancy BMI category	
Underweight	0 (0%)
Normal weight	80 (27.3%)
Overweight	163 (55.6%)
Obesity	50 (17.1%)
Pre‐pregnancy BMI	24.30 ± 2.99 (18.08–36.85)
Anxiety score	70.44 ± 16.07 (34.00–119.00)
Diet quality score	42.76 ± 9.73 (20.00–72.00)
Dietary iron adequacy (%)	44.08 ± 17.71 (8.50–86.06)
Current gestational weight gain (GWG) (kg)	7.47 ± 3.49 (−1.30–20.00)
Parity, *n* (%)	
Once	151 (51.5%)
Twice or more	142 (48.5%)

*Note:* Data are presented as mean ± SD (minimum–maximum) or *n* (%). Anxiety score was assessed using the Pregnancy‐Related Anxiety Scale (PRAS; score range: 33–132), with higher scores indicating greater pregnancy‐related anxiety. Diet quality score was assessed using the Dietary Quality Index for Pregnancy (DQI‐P; score range: 0–80), with higher scores indicating better diet quality.

Abbreviations: BMI, body mass index; GWG, gestational weight gain; SD, standard deviation.

The multiple linear regression model included maternal age, pre‐pregnancy BMI, education level, income level, trimester, and planned pregnancystatus (Table 2). The multiple linear regression analysis indicated that the model was statistically significant (F [12,280] = 8.16, *p* < 0.001) and explained 23% of the variance in PRAS scores (Adjusted *R* ^2^ = 0.230). Maternal age (*B* = −0.825, *p* < 0.001), income level (*B* = −6.565, *p* < 0.001), and planned pregnancy status (*B* = 8.744, *p* < 0.001) were significantly associated with PRAS scores. However, PDQI, dietary iron adequacy (%), pre‐pregnancy BMI, trimester, and education level did not show significant associations with PRAS scores (*p* > 0.05). Additionally, all VIF values were below 2, indicating no evidence of multicollinearity within the model. As a sensitivity analysis, the moderation model was repeated using categorical pre‐pregnancy BMI instead of continuous BMI. The findings were essentially unchanged, with the interaction between PDQI and dietary iron adequacy remaining statistically significant (*β* = −0.0158, *p* = 0.005; data not shown).

**TABLE 2:** Multiple linear regression analysis predicting anxiety.

Predictor	*B*	SE	*β*	*t*	*p*
PDQI	−0.097	0.100	−0.059	−0.977	0.330
Dietary iron adequacy (%)	0.048	0.056	0.053	0.867	0.387
Pre‐pregnancy BMI	0.253	0.319	0.047	0.794	0.428
Maternal age	−0.825	0.217	−0.221	−3.795	**< 0.001**
Trimester	0.321	1.458	0.012	0.220	0.826
Education	1.303	0.933	0.081	1.397	0.163
Income	−6.565	1.578	−0.241	−4.161	**< 0.001**
Planned pregnancy	8.744	1.889	0.255	4.629	**< 0.001**

*Note:* Model fit: *R* ^2^ = 0.262, Adjusted *R* ^2^ = 0.230, F (12, 280) = 8.16, *p* < 0.001, Durbin–Watson = 1.65. *B* values represent unstandardized regression coefficients, and *β* values represent standardized coefficients. The model was adjusted for maternal age, pre‐pregnancy BMI, trimester, education, income, and planned pregnancy. No issues with multicollinearity were observed (VIF < 2). Bold values indicate statistically significant *p*‐values (p < 0.05). All tests were two‐tailed, and statistical significance was set at *p* < 0.05.

Abbreviations: β, Standardized coefficient; B, Unstandardized regression coefficient; BMI, Body Mass Index; CI, Confidence Interval; PDQI, Diet Quality Index for pregnancy; PRAS (dependent variable), Pregnancy‐related Anxiety Scale; SE, Standard error; VIF, Variance Inflation Factor.

A moderation analysis revealed a significant interaction between PDQI and dietary iron adequacy (%) (*B* = −2.501, *p* = 0.007). The overall model explained 25.8% of the variance in PRAS scores (*R* ^2^ = 0.258, *p* < 0.001). Although PDQI was significantly associated with dietary iron adequacy (%) (*p* < 0.001), dietary iron adequacy (%) was not associated with PRAS scores (*p* = 0.246) (Table 3).

**TABLE 3:** Moderation analysis examining the interaction between diet quality and iron percentage in predicting anxiety (*N* = 293).

Predictor	*B*	SE	*β*	*t*	*p*
Z‐PDQI	−1.567	0.961	−0.098	−1.630	0.104
Z‐Dietary iron adequacy (%)	1.169	0.950	0.073	1.230	0.220
**Diet × Iron**	**−2.501**	0.916	**−0.147**	**−2.730**	**0.007**
Maternal age	−0.770	0.211	−0.206	−3.656	**< 0.001**
Income	−5.822	1.550	−0.213	−3.756	**< 0.001**
Planned pregnancy	7.725	1.858	0.225	4.158	**< 0.001**
Other covariates	—	—	—	—	ns

*Note:* Model fit: *R* ^2^ = 0.258; Adjusted *R* ^2^ = 0.231; F (10,282) = 9.792; *p* < 0.001. *B* values represent unstandardized regression coefficients. *β* values represent standardized regression coefficients. Z‐Diet Quality and Z‐Dietary iron adequacy (%) indicate standardized (z‐transformed) variables used to reduce multicollinearity in the interaction model. The interaction term (diet × iron) represents the product of standardized diet quality and iron percentage variables. The model was adjusted for maternal age, income level, planned pregnancy status, and other obstetric covariates. *R* ^2^ represents the proportion of variance in anxiety scores explained by the model. All tests were two‐tailed and statistical significance was set at *p* < 0.05. Bold values indicate statistically significant *p*‐values (*p* < 0.05). The Diet × Iron interaction term is highlighted to indicate the significant moderating effect of dietary iron adequacy on the association between diet quality and pregnancy‐related anxiety.

Abbreviations: β, Standardized coefficient; B, Unstandardized regression coefficient; ns, not significant; PDQI, Diet Quality Index for pregnancy; SE, Standard error; *t*, *t* statistic.

Simple slope analysis indicated that the protective association between diet quality and anxiety was only statistically significant among women with high dietary iron adequacy (+1 SD above the mean): higher PDQI scores were significantly associated with lower PRAS scores in this group (*B* = −3.510, SE = 1.479, *t* = −2.374, *p* = 0.018, 95% CI [−6.409, −0.612]) (Table 4). At mean levels of dietary iron adequacy, the association was negative but non‐significant (*B* = −1.051, SE = 0.977, *p* = 0.283). Among women with low dietary iron adequacy (−1 SD), the association was positive and non‐significant (*B* = 1.409, SE = 1.202, *p* = 0.242), indicating that the anxiolytic benefit of a high‐quality diet was absent in this group.

**TABLE 4:** Simple slope analysis: Conditional effects of prenatal diet quality on pregnancy‐related anxiety at three levels of dietary iron adequacy (*N* = 293).

Predictor	*B*	SE	*t*	*p*	95% CI
Low dietary iron adequacy (−1 SD)	1.409	1.202	1.172	0.242	−0.947, 3.765
Mean dietary iron adequacy	−1.051	0.977	−1.076	0.283	−2.965, 0.864
High dietary iron adequacy (+1 SD)	**−3.510**	**1.479**	**−2.374**	**0.018**	**−6.409, −0.612**

*Note:* *B* values represent unstandardized simple slope coefficients reflecting the association between PDQI and PRAS scores at each level of dietary iron adequacy. Low and high iron adequacy were defined as ±1 SD from the mean. Bold values indicate statistical significance at *p* < 0.05.

Abbreviations: CI, confidence interval; PDQI, Prenatal Diet Quality Index; PRAS, Pregnancy‐Related Anxiety Scale; SD, standard deviation.

**FIGURE 1:**
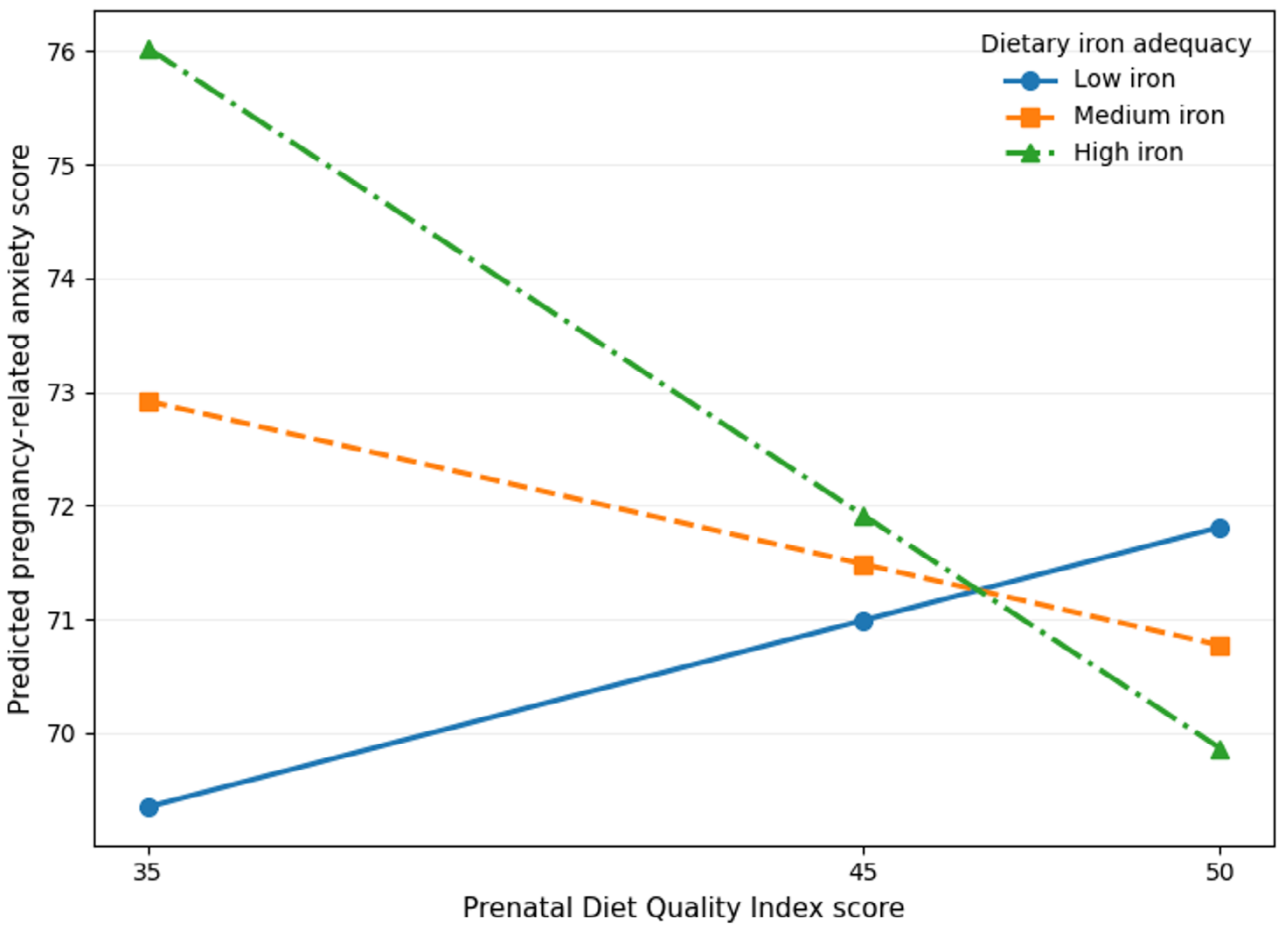
Interaction between diet quality and iron adequacy in predicting pregnancy‐related anxiety scores. Predicted pregnancy‐related anxiety scores were estimated from a moderation analysis using PROCESS macro version 4.2 (Model 1) with HC3 heteroscedasticity‐consistent standard errors, adjusting for income, education, trimester, maternal age, and pre‐pregnancy BMI. Predicted values are presented at the 16th, 50th, and 84th percentiles of dietary iron adequacy. Pregnancy‐related anxiety was assessed using the Pregnancy‐Related Anxiety Scale (PRAS; range: 33–132), with higher scores indicating greater pregnancy‐related anxiety. Prenatal diet quality was assessed using the Prenatal Diet Quality Index (PDQI), where higher scores indicate better diet quality.

Figure 1 illustrates the interaction between PDQI and dietary iron adequacy (%) in predicting PRAS scores. Among women with relatively high dietary iron adequacy (+1 SD; approximately 61.79% of the RDA), higher diet quality was significantly associated with lower predicted anxiety scores. At mean dietary iron adequacy, higher PDQI scores were associated with a slight, non‐significant decrease in predicted PRAS scores. In contrast, among women with low dietary iron adequacy (−1 SD; approximately 26.37% of the RDA), higher PDQI scores were not associated with lower anxiety; instead, a slight, non‐significant increase in predicted PRAS scores was observed.

## 4 Discussion

To date, no studies have examined whether dietary iron adequacy moderates the association between prenatal diet quality and pregnancy‐related anxiety in pregnant women in Türkiye. Examining this moderating effect is important because a high‐quality dietary pattern may not provide the same psychological benefits when the intake of a key micronutrient is inadequate. This information may help explain heterogeneity in previous findings on diet quality and maternal mental health (Fowles et al. 2012; Nathanson et al. 2018; Wang et al. 2023) and may inform whether antenatal dietary counseling should consider nutrient‐specific adequacy alongside overall diet quality (Cano‐Ibáñez et al. 2020; Georgieff 2020). In this cross‐sectional sample, prenatal diet quality was not independently associated with pregnancy‐related anxiety; however, its association appeared to vary according to dietary iron adequacy. This suggests that the nutritional context in which overall diet quality is achieved may matter for maternal psychological well‐being. The fact that the interaction coefficient is negative and statistically significant (Diet × Iron: *B* = −2.501, *p* = 0.007) indicates that as the percentage of dietary iron adequacy increases, the increase in PDQI becomes associated with a more pronounced decrease in anxiety scores. Conversely, when iron levels are low, the relationship between PDQI and PRAS scores weakens and may even reverse. This finding suggests that not only overall dietary quality, but also dietary iron adequacy may be a critical contextual variable for psychological well‐being during pregnancy.

The literature reports heterogeneous results regarding the relationship between iron status during pregnancy and psychological symptoms. A long‐term study conducted among pregnant women in Ghana found that improvements in hemoglobin and ferritin levels from the first to the second trimester were associated with a reduction in depressive/anxiety symptoms and improvements in various areas of quality of life. The idea that better iron status in early pregnancy may contribute to the development of psychological well‐being has been supported (Pobee et al. 2024). On the other hand, in the FinnBrain Birth Cohort, no significant association was found between gestational anemia (Hb < 11 g/dL) and prenatal/postpartum depression; however, a weak association with anxiety in early pregnancy and an association with late pregnancy anxiety when using a lower hemoglobin threshold (< 10 g/dL) were reported (Kemppinen et al. 2022). These findings suggest that the iron‐anxiety relationship may vary depending on the gestational age, severity of anemia, and psychological measurement tool used, rather than being a strong and unidirectional relationship. In this context, the PDQI × iron interaction observed in our study indicates that PRAS may be more accurately assessed in the context of dietary quality rather than iron levels alone. However, hemoglobin, ferritin, transferrin saturation, or other biochemical indicators of iron status were not available in the present study. Therefore, we could not determine the prevalence or severity of anemia or iron deficiency among the participants, and our findings cannot be directly compared with studies based on biomarker‐confirmed iron status.

However, dietary iron adequacy in the present study was estimated solely from dietary intake and therefore may not fully reflect actual iron status. In Türkiye, the Ministry of Health has implemented a nationwide iron supplementation program recommending routine iron supplementation for all pregnant women from the fourth month of pregnancy until 3 months postpartum (a total of 9 months) (Republic of Türkiye Ministry of Health 2007). Consistent with this policy, studies conducted in Türkiye have reported that iron supplement use during pregnancy is common, with 58.1%–75.6% of pregnant women reporting the use of iron‐containing supplements (Kabalcıoğlu Bucak and Kartal 2026). Moreover, Çıtıl et al. (2014) reported a significantly lower prevalence of anemia among women using iron–multivitamin supplements compared with non‐users. Therefore, some participants in our study may have had better iron status than suggested by dietary intake alone. Consequently, dietary iron adequacy should be interpreted as an indicator of dietary iron intake rather than a direct measure of physiological iron status, and this should be considered when interpreting the present findings.

Notably, at lower levels of dietary iron adequacy, the association between prenatal diet quality and pregnancy‐related anxiety was weak or absent, whereas at relatively higher levels of dietary iron adequacy, higher PDQI scores were associated with lower PRAS scores. Simple slope analysis showed that this inverse association was statistically significant only at +1 SD above the mean dietary iron adequacy. However, the term “high dietary iron adequacy” should be interpreted relatively within this sample. Given the low mean dietary iron adequacy of 44.08% of the RDA, the +1 SD level corresponded to approximately 61.79% of the RDA and therefore did not represent achievement of the recommended intake or confirm that these women were iron‐replete. Accordingly, the moderation finding indicates that the association between higher diet quality and lower anxiety was more apparent among women with comparatively greater dietary iron intake within this generally low‐intake sample, rather than among women with objectively adequate iron intake or confirmed sufficient iron status. These findings suggest that the potential psychological benefits of a high‐quality diet during pregnancy may depend on achieving adequate iron intake.

One possible explanation for this finding is that iron plays a critical role in neurotransmitter synthesis and brain energy metabolism. Iron is required for enzymes involved in the synthesis of monoamine neurotransmitters such as serotonin and dopamine (Kulaszyńska et al. 2024), which are closely related to mood and anxiety regulation (Leung and Kyung 2024). In addition, iron is essential for mitochondrial function and ATP production, which are necessary for optimal brain function (Spence et al. 2020). Therefore, even when overall diet quality is high, inadequate iron intake may limit the potential psychological benefits of a healthy dietary pattern. Nevertheless, the observed interaction may not reflect an effect specific to iron. Dietary iron adequacy may also act as a marker of broader micronutrient adequacy or the consumption of nutrient‐dense foods that provide folate, vitamin B12, zinc, protein, and other nutrients relevant to neurological functioning (Parrott et al. 2009; Kangalgil et al. 2022). Moreover, iron was the only nutrient‐specific moderator examined in this study. Future studies should assess multiple micronutrients simultaneously to determine whether the observed association is specific to iron or reflects overall micronutrient adequacy.

The inclusion of biochemical iron‐status measures could change the observed relationship because dietary intake does not necessarily reflect iron stores, absorption, or physiological requirements (Georgieff 2020; Kangalgil et al. 2022). Similarly, iron supplementation may weaken or alter the association between dietary iron intake and anxiety, as women with inadequate dietary intake may still have adequate iron status through supplementation (Nair et al. 2004). Future studies should therefore assess dietary iron intake together with supplement type, dose, duration, adherence, hemoglobin, and ferritin concentrations.

It is also important to note that non‐significant main effects are not uncommon in interaction models. The main effects represent the association between PDQI and anxiety at the average level of the moderator (in this case, the average iron intake level due to standardization), whereas the strength and direction of this association may vary at different levels of iron intake. Therefore, the present findings suggest that a “one‐size‐fits‐all” nutrition approach may be insufficient for psychological outcomes in pregnant women, and that improvements in diet quality may provide greater psychological benefits particularly among women who achieve adequate iron intake.

One possible biological explanation for this interaction is the role of iron in neuropsychiatric functions. Iron is associated with oxygen transport and energy metabolism, as well as neurotransmitter synthesis and nervous system functions (Kim and Wessling‐Resnick 2014). Increased iron requirements during pregnancy and decreased iron stores may be associated with symptoms such as fatigue, decreased cognitive performance, and mood swings (Georgieff 2020; Greig et al. 2013). In this context, when iron sufficiency is ensured, a more balanced macro/micronutrient profile and anti‐inflammatory dietary pattern resulting from high dietary quality may have a more pronounced protective effect on anxiety symptoms. In the case of iron deficiency, even if overall dietary quality improves, psychological benefits may be limited due to iron‐specific physiological constraints. In a rat study, anxiety‐like behaviors, anhedonia‐like responses, and changes in brain monoamines were observed in offspring born to mother rats fed an iron‐deficient diet during pregnancy and lactation and continued on an iron‐deficient diet during the postnatal period (Kemp et al. 2024). Although these findings do not directly indicate anxiety in pregnant women, they suggest that nutritional components may jointly affect neuropsychiatric processes, providing a biologically plausible framework for the PDQI × iron interaction observed in our study.

From a public health perspective, fortified and functional foods may provide complementary strategies for improving micronutrient intake in populations at risk of nutritional inadequacy. Recent developments in food fortification and biofortification may improve nutrient stability, bioavailability, and delivery; however, nutrient interactions, the risk of excessive intake, affordability, and unequal access should also be considered (Hossain et al. 2025). In pregnant women, iron‐fortified foods may therefore represent a supportive approach when habitual dietary intake is insufficient, but they should complement rather than replace individualized dietary assessment, biomarker‐based evaluation, and guideline‐based supplementation.

Interestingly, anxiety scores were higher among women with planned pregnancies. Previous research suggests that women who actively plan their pregnancies may experience increased expectations and a heightened sense of responsibility, which can paradoxically elevate pregnancy‐related anxiety (Maghalian et al. 2024; Nelson et al. 2022). This situation may be related to increased anxiety levels specific to pregnancy in some women experiencing planned pregnancies due to a tendency to exercise greater control over the pregnancy process, heightened expectations, and a sense of responsibility.

In the adjusted model, maternal age, income level, and planned pregnancy were significant correlates of anxiety (all *p* < 0.001), which supports the role of sociodemographic and obstetric characteristics in pregnancy‐related anxiety. More broadly, maternal health outcomes and healthcare‐related decisions are shaped by interacting sociodemographic, obstetric, and healthcare‐system factors. For example, a hospital‐based study in Bangladesh found that maternal age, place of residence, family structure, previous cesarean delivery, maternal preference, and physician influence were associated with the mode of delivery (Hossain et al. 2024). Although mode of delivery is distinct from pregnancy‐related anxiety, these findings illustrate the broader contextual and multifactorial nature of maternal health outcomes.

In our analysis, income level independently predicted anxiety scores but was not significantly associated with diet quality. Therefore, our findings do not allow us to conclude that the association between income and anxiety operates through diet quality. The socioeconomic context may also shape the ability to achieve a diverse and nutritionally adequate diet. In a community‐based study conducted in rural Southwestern Bangladesh during the COVID‐19 pandemic, household income, education, occupation, family size, and knowledge of a balanced diet were significantly associated with household dietary diversity (Shuvo et al. 2025). Although that study assessed household‐level dietary diversity under pandemic conditions rather than pregnancy‐specific diet quality, its findings reinforce the importance of considering socioeconomic access and nutritional knowledge when interpreting dietary adequacy. While income level was found to independently predict anxiety scores, it was not significantly associated with diet quality (high PDQI status) in our analysis. Therefore, our findings do not allow us to conclude directly that the association between income and anxiety operates through diet quality. Nevertheless, given prior evidence linking socioeconomic disadvantage with prenatal dietary quality and maternal psychological well‐being, this hypothesis remains plausible and warrants testing in larger cohorts using mediation‐based approaches (Biete et al. 2025; Wang et al. 2023). Notably, the PDQI × iron interaction retained statistical significance after adjustment, indicating that this nutritional interaction may explain variance in anxiety that is not accounted for by sociodemographic and obstetric factors. However, the explanatory power of the model is moderate (*R* ^2^ = 0.258), so the multifactorial nature of anxiety should be considered; the role of additional determinants such as psychosocial stressors, social support, sleep, physical activity, and pregnancy complications should be more comprehensively evaluated in future studies.

One of the strengths of this study was the combined use of two pregnancy‐specific assessment tools. Pregnancy‐related anxiety was assessed using the PRAS, which was originally developed and validated by Brunton et al. (2019), and its Turkish validity and reliability were established by Şolt Kırca and Kanza Gül (Şolt and Gül 2020). Prenatal diet quality was assessed using the PDQI, a pregnancy‐specific dietary index developed by Bodnar and Siega‐Riz (2002). Including the interaction term (PDQI × dietary iron adequacy) in the model allows for a more nuanced interpretation that the diet‐anxiety relationship is not unidirectional and homogeneous; it may vary depending on contextual factors such as iron status. Moreover, simple slope analysis was conducted to formally test the significance of the interaction at each level of the moderator, thereby enhancing the interpretability and clinical applicability of the moderation findings.

However, the findings should be interpreted within certain limitations. First, because the study was cross‐sectional, causal inferences cannot be made; the possibility of reverse causality, namely whether diet quality affects PRAS scores or higher anxiety alters food choices, cannot be ruled out. Second, because the food consumption data underlying the PDQI were self‐reported, measurement error and recall bias are possible; similarly, because PRAS scores were also self‐reported, social desirability bias may have influenced the findings. Third, the moderator reflected dietary iron adequacy calculated from food intake rather than biomarker‐confirmed iron status or total iron exposure from foods and supplements. Therefore, the observed interaction should be interpreted as a dietary‐context finding rather than evidence of physiological iron sufficiency or an iron‐specific effect. Information on the type, dose, duration, and adherence to iron supplementation was not incorporated into the moderation analysis. In addition, gestational weight gain was calculated using pre‐pregnancy weight and weight recorded at a single assessment conducted at different stages of pregnancy. Therefore, this variable represents weight gain up to the time of assessment rather than total gestational weight gain and may not be directly comparable across trimesters. Standardized variables were used in the interaction model to reduce multicollinearity; simple slope analysis was subsequently conducted to provide clinically interpretable effect sizes at each level of the moderator. Finally, although some covariates were included in the model, the lack of measurement of important variables such as sleep quality, physical activity, social support, previous psychiatric history, pregnancy complications, and stress levels may lead to residual confounding; therefore, the results should be interpreted with caution.

## 5 Conclusion

This study suggests that diet quality alone may not be sufficient to influence psychological outcomes during pregnancy unless micronutrient adequacy is achieved. In particular, adequate iron intake may be a key nutritional factor that enables the potential psychological benefits of a high‐quality diet to emerge. These findings highlight the importance of integrating micronutrient adequacy into dietary counseling strategies during pregnancy rather than focusing solely on overall diet quality. Future longitudinal and biomarker‐based studies are needed to clarify the causal relationship between diet quality, micronutrient status, and maternal mental health.

## Author Contributions

**Beyza Sisman:** investigation, writing – original draft. **Lara Hidimoglu:** investigation, writing – original draft. **Ezgi Arslan Yuksel:** writing – original draft, writing – review and editing, methodology, conceptualization, resources, formal analysis. **Ahmet Murat Gunal:** supervision. **Tuba Kahraman:** writing – original draft, resources, writing – review and editing.

## Funding

The authors have nothing to report.

## Conflicts of Interest

The authors declare no conflicts of interest.

## Data Availability

Due to legal restrictions and the need to protect participant confidentiality, the data supporting the findings of this study are not publicly available. However, the data can be made available from Ezgi Arslan Yüksel upon reasonable request and with appropriate permissions.
